# Comparative analysis of foam-only versus carbon-plated advanced footwear technology spikes in distance runners

**DOI:** 10.3389/fphys.2025.1703854

**Published:** 2025-11-19

**Authors:** Yiwei Wu, Haoran Zhang, Shuhan Wang, Changda Lu, Qingjun Xing, Yinshen Tian, Dianchen He, Lixin Sun, Yanfei Shen

**Affiliations:** 1 School of Sport Science, Beijing Sport University, Beijing, China; 2 AI Sports Engineering Lab, School of Sports Engineering, Beijing Sport University, Beijing, China; 3 Sports Data Center of China, Beijing Sport University, Beijing, China; 4 Physical Education Department, Shenyang Sport University, Shenyang, China; 5 Key Laboratory for Performance Training and Recovery of General Administration of Sport, Beijing Sport University, Beijing, China; 6 Engineering Research Center of Strength and Conditioning Training Key Core Technology Integrated System and Equipment, Ministry of Education, Beijing Sport University, Beijing, China

**Keywords:** advanced footwear technology, spikes, carbon fiber plate, distance running, running economy

## Abstract

**Background:**

Advanced footwear technology (AFT) spikes are commonly offered in two configurations: foam only and foam combined with a carbon fiber plate. Whether the plate provides additional metabolic or performance benefits over foam-only designs remains uncertain. Therefore, this study compared physiological, biomechanical, and perceptual responses to two commercially available AFT spikes (Nike ZoomX Dragonfly, foam only; Nike Air Zoom Victory, foam plus carbon plate) in trained and national-level distance runners.

**Methods:**

Thirteen male middle- and long-distance runners (trained, n = 6; national-level, n = 7) completed three randomized 1600-m submaximal trials on an outdoor track at 16 km·h^−1^ (trained) or 18 km·h^−1^ (national-level). Running economy (RE) was assessed using a portable gas analyzer (MetaMax 3B-R2); spatiotemporal gait variables were recorded with shoe-mounted sensors (RunScribe^TM^); and participants rated comfort, cushioning, and perceived performance on a 10-point Likert scale.

**Results:**

In the national-level group, both foam-only spikes (Dragonfly1, Dragonfly2) produced better RE than the carbon-plated model (Victory1), with no difference between the two foam-only versions. In the trained group, RE did not differ across spikes. Energetic cost paralleled the VO_2_ findings. For gait parameters, stride length and step frequency were unchanged across conditions in both groups. Whereas contact time in the national-level group was longer in Dragonfly1 than in Dragonfly2 and Victory1, whereas Dragonfly2 and Victory1 did not differ; in the trained group, contact time was unchanged across spikes. Subjectively, across all participants, foam-only spikes were rated more comfortable and more cushioned, whereas perceived performance did not differ between models.

**Conclusion:**

At long-distance race paces, foam-only AFT spikes improved RE and were perceived as more comfortable than a plate-integrated spike in national-level athletes. Adding a carbon plate did not guarantee a metabolic benefit and may increase energetic cost when shoe stiffness exceeds an athlete-specific optimum. Spike selection, particularly for track events, should demystify carbon plates and prioritize the individualized selection of shoe stiffness and geometry matched to event distance, running speed, and athlete-specific biomechanics.

## 1 Introduction

In competitive middle- and long-distance running, footwear can influence performance by reducing metabolic cost (Fuller et al., 2015; Xu et al., 2025) and improving running economy (RE) (Burns and Joubert, 2024; Sinclair et al., 2014). Even a 1.1% reduction in metabolic cost may yield nearly a 0.8% improvement in 3000 m performance among trained runners (Hoogkamer et al., 2016), emphasizing the performance relevance of small energetic gains. Over the past decades, the emergence of advanced footwear technology (AFT) has introduced substantial changes to competitive distance running and become widely adopted by elite athletes across various events (Senefeld et al., 2021; Rodrigo-Carranza et al., 2022; Goss et al., 2022). These AFT shoes typically combine lightweight construction, compliant and resilient foams (e.g., PEBA), enhanced rocker geometry, and a stiff embedded element (e.g., curved carbon fiber plate) to increase longitudinal bending stiffness (LBS) (Carranza, 2023).

Multiple studies have confirmed that AFT shoes significantly improve RE and long-distance running performance compared to traditional racing shoes (Barnes and Kilding, 2019; Hunter et al., 2019; Joubert and Jones, 2022; Joubert et al., 2023). These improvements in RE help athletes sustain faster running speeds at a given physiological intensity, which is typically reflected by a fixed oxygen uptake or metabolic power, and may explain much of the performance gains observed in competitive distance events (Rodrigo-Carranza et al., 2023; Ruiz-Alias et al., 2024; Castellanos-Salamanca et al., 2024; Ortega et al., 2021). Building on these findings in road footwear, the application of AFT has extended from road racing shoes to track spikes, contributing to notable improvements in mid- and long-distance track performances (Healey et al., 2022; Willwacher et al., 2023). Track implementations apply the same design principles: low mass, compliant foams, and, in some models, a curved carbon-fiber plate used to tune LBS. The geometry is adapted to the stack-height limits and the forefoot-loaded mechanics of spikes. Consequently, AFT spikes generally fall into two structural categories: foam-only designs with compliant, resilient midsoles, and foam-plus-plate designs that incorporate a carbon-fiber plate. However, it remains unclear whether the addition of a carbon plate consistently confers metabolic or performance advantages over foam-only models, particularly in events ranging from 1,500 m to 10,000 m. Clarifying this distinction is essential for understanding how specific footwear design elements contribute to RE and for guiding evidence-based spike selection across different performance levels. While several recent studies have compared AFT spikes with and without carbon plates (Rodrigo-Carranza et al., 2025; Alda-Blanco et al., 2024), controlled laboratory comparisons remain limited, particularly under standardized submaximal protocols, as was done previously with AFT road racing shoes (Knopp et al., 2023; Healey and Hoogkamer, 2022; Rodrigo-Carranza et al., 2024).

Recent empirical evidence has consistently demonstrated that AFT spikes enhance RE and performance relative to conventional models. Across a range of controlled and field-based investigations, improvements of approximately 2%–5% in RE and 2%–3% in race performance have been observed (Joubert et al., 2024; Rodrigo-Carranza et al., 2025; Bertschy et al., 2025). These performance gains appear to be modulated by running speed and shoe configuration, with mechanical adaptations at higher speeds not always corresponding to the metabolic improvements typically seen at submaximal intensities (Needles and Grabowski, 2024; Russo et al., 2022). Despite the growing body of evidence supporting the benefits of AFT spikes, comparative analyses of different AFT spike architectures, particularly those that isolate the effects of foam versus plate elements under standardized laboratory conditions, remain scarce. This limitation leaves important questions unanswered regarding model-specific performance mechanisms and optimal spike selection for diverse athlete populations.

The primary aim of this study was to compare, under submaximal running conditions, the effects of two commercially available AFT spike models on RE in trained and national-level distance runners: a foam-only design and a design that combines foam with a carbon-fiber plate. A secondary aim was to assess spatiotemporal gait variables (stride frequency, stride length, and contact time) and perceptual outcomes (comfort, cushioning, and perceived performance enhancement) to provide a comprehensive comparison of the two configurations (Hébert-Losier et al., 2024). We hypothesized that (H1) at distance-relevant submaximal speeds (16–18 km·h^−1^), the foam-only spikes would elicit lower oxygen uptake and energetic cost (i.e., better RE) than the foam-plus-plate spikes, with a larger effect in national-level athletes; (H2) relative to foam-only, the foam-plus-plate spikes would be associated with shorter contact time and higher step frequency, with no systematic difference in stride length; and (H3) perceptual ratings would favor foam-only for comfort and cushioning, whereas perceived performance enhancement would be rated higher for the foam-plus-plate spikes.

## 2 Materials and methods

### 2.1 Participants

Thirteen male distance runners (ages: 21.6 ± 1.8 years; height: 175.5 ± 3.6 cm; body mass: 60.8 ± 5.6 kg) participated in this study. The inclusion criteria were the following: 1) Participants must be able to perform experimental procedures independently and not have had any musculoskeletal or chronic neurological disorders in the past year; 2) fitting a men’s 39-41 EU shoe size; 3) requirements in 800 m and above middle and long-distance running events to reach China’s national second level athlete performance as described later; and 4) all participants had previously been spikes and AFT spikes users. Participants were divided into two groups based on their personal best performances in middle- and long-distance track events, according to the classification standards issued by the General Administration of Sport of China and the performance-level framework proposed by McKay et al. (2021). The national-level group (n = 7) included athletes whose best times met or exceeded first-class standards (e.g., 1:54.50 for 800 m, 3:54.90 for 1500 m, 8:35.00 for 3000 m, 14:40.00 for 5000 m, and 30:50.00 for 10,000 m). The trained group (n = 6) comprised athletes whose performances corresponded to second-class standards (e.g., 2:02.11 for 800 m, 4:13.86 for 1500 m, 9:10.00 for 3000 m, 15:55.72 for 5000 m, and 33:30.09 for 10,000 m). All race times are presented in min:s format. Utilizing effect sizes (1.178) previously seen in AFT shoes (Knopp et al., 2023), an *a priori* power analysis (G*Power, version 3.1) revealed that a sample size of 6 participants would be adequate to achieve a power of 0.8 with an α of 0.05.

All participants signed an informed consent form before participating in the study. The study was conducted in accordance with the Declaration of Helsinki and approved by the Beijing Sport University Ethics Committee (2024491H).

### 2.2 Spikes characteristics

**FIGURE 1:**
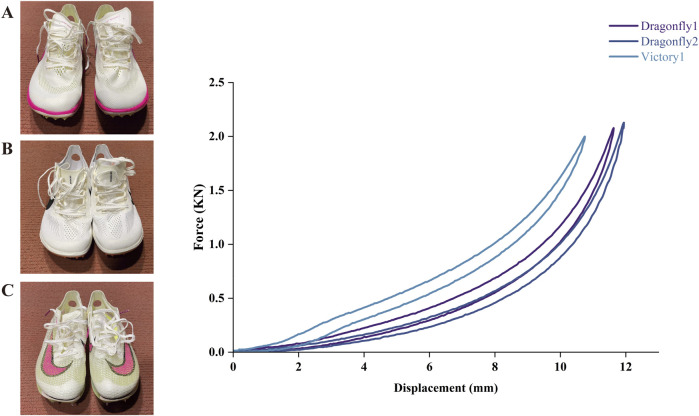
Force-deformation curves, and energy return metrics for each AFT spike during vertical forefoot loading with a peak force of 2000N and contact time of 200 ms. As vertical force is applied, the shoe forefoot deforms (upper trace in each graph). When the shoe is unloaded, the force returns to zero as the forefoot recoils (lower trace in each graph). The area between the loading and unloading curves reflects the mechanical energy lost as heat, while the area beneath the lower trace represents the amount of elastic energy returned. **(A)** Nike ZoomX Dragonfly1 (Nike Inc., United States), **(B)** Nike ZoomX Dragonfly2 (Nike Inc., United States) and **(C)** Nike Air Zoom Victory1 (Nike Inc., United States).

Throughout the experimental protocol, analyzed shoe conditions included three commercially available AFT spikes that differed in their mass, forefoot LBS, and energy return. Specifically, we tested an AFT model with modern foam (Nike ZoomX Dragonfly1 and Dragonfly2, later iteration with minimal design changes), and an AFT spike model with a curved carbon fiber plate (Nike Air Zoom Victory1), as shown in Figure 1. All AFT spikes were equipped with standardized 6 mm pins to ensure consistent traction. The full footwear lineup and specifications are reported in Table 1, and all spikes were new before the start of this study. Participants could observe the spikes but had no opportunity to manipulate them and remained unaware of differences in spike properties.

**TABLE 1:** Descriptive characteristics of the three AFT spikes.

	Mass (g)	Forefoot thickness (mm)	Heel thickness (mm)	Forefoot LBS (N/mm)	Forefoot energy return (%)
Dragonfly1	121	17.8	21.1	13.4	82.1
Dragonfly2	117	18.3	20.8	12.8	83.7
Victory1	119	19.8	19.5	41.2	83.9

In accordance with World Athletics regulations, sole thickness was measured at the center of the forefoot and heel. Specifically, the forefoot thickness was determined at 75% of the shoe’s internal length from the heel, while heel thickness was measured at 12% of internal length. LBS was assessed in accordance with the national standard (GB/T 32023: Footwear—Test methods for whole shoes—Rigidity of flexing area). A three-point bending configuration was used, with each spike placed on two support points spaced 80 mm apart. A vertical force was applied to the widest portion of the forefoot using an Instron Universal Testing Machine (model 3345, Instron, Norwood, MA, United States) at a displacement rate of 150 mm/s to a maximum displacement of 14.5 mm. Force–displacement data were recorded between 5.8 and 9.7 mm and used to calculate LBS. Each shoe was tested three times, and the mean stiffness value was used in subsequent analysis. Energy return was evaluated in accordance with the national standard GB/T 38012: Footwear—Test methods for whole shoe—Impact shock attenuating property. Testing was performed using a servo-hydraulic testing system (Instron 8800 Series, Norwood, MA, United States). Each shoe sample underwent 30 consecutive impact cycles with a target peak force of 2000 N and an impact contact time of 200 ms. During each cycle, force and displacement data were continuously recorded. The absorbed energy was defined as the difference between the energy input during the loading phase and the energy recovered during unloading. Following the standard protocol, the 30th impact was used to represent the energy return value for analysis.

### 2.3 Experimental protocol

We evaluated the impact of three AFT spikes on RE using a randomized crossover experimental design to avoid any order effects. All experiments of this study took place at the first runway of a standard outdoor 400 m synthetic rubber track and field stadium during the daytime. To minimize potential VO_2_ variations due to circadian rhythms, each participant visited the experimental setting once, and we conducted all data collection sessions at the same time of day. Prior to beginning the RE testing trials, all participants completed 10 min of jogging as a warm-up in their own shoes at a self-selected pace. All participants then completed 3 repetitions of 1600 m running trials at 16 km·h^−1^ for the group of trained runners and 18 km·h^−1^ for the group of national runners with each type of AFT spike condition (total 3 × 1600 m). The order of footwear testing was randomized individually for each participant using a simple randomization procedure and minimizing potential order effects. The selected running intensities were below the second ventilatory threshold to ensure that steady-state VO_2_ could be achieved for RE measurement. Meanwhile, to ensure that participants maintained a constant pace during the RE test, we used photoelectric timing gates with an accuracy of 0.001 s arranged at both ends of the track and field, and they were given an acoustic signal each 200 m. At least five minutes of rest were allowed between trials to change shoes and allow physiological recovery. This recovery duration has been shown to be sufficient for restoring steady-state VO_2_ between repeated submaximal running bouts in similar protocols (Ruiz-Alias et al., 2024). All footwear trials for each participant were conducted within a single testing session on the same day, ensuring comparable environmental conditions within individuals (controlled environmental conditions [50 m altitude, 17 °C–24 °C, 41%–69% relative humidity]).

### 2.4 Measurements

During the RE test, oxygen consumption and carbon dioxide production were continuously recorded throughout each trial using a calibrated MetaMax 3B-R2 system (Cortex Biophysic, Leipzig, Germany). Before each testing session, we calibrated this system according to the manufacturer’s instructions. We monitored each participant’s respiratory exchange ratio (RER) throughout each trial to ensure that everyone primarily relied on aerobic metabolism during running, indicated by RER ≤1.0 (Brooks et al., 2005; Barnes and Kilding, 2015). RE was then calculated using the average VO_2_ and VCO_2_ values from the final 2 min of each trial, after participants reached a physiological steady state. The oxygen consumption between the penultimate and final minute of each 1600 m bout differed by less than 0.5% (Rodrigo-Carranza et al., 2023), confirming that metabolic steady state was achieved for all participants. RE was expressed as both VO_2_ (mL/kg/min) and energetic cost (W/kg), which was calculated from VO_2_ and VCO_2_ values using the Péronnet and Masicotte equation (Peronnet and Massicotte, 1991).

**FIGURE 2:**
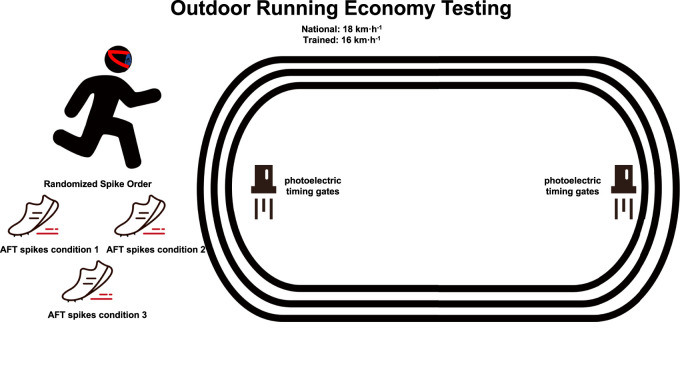
Illustration of the methods protocol of the present study. Schematic representation of the experimental protocol. Participants performed RE trials in three types of AFT spikes on a 400 m outdoor track. Each condition involved three 1600 m trials at fixed speeds (16 km/h for trained runners, 18 km/h for national-level runners), followed by a standardized warm-up. Running speed was monitored using photoelectric timing gates placed 200 m apart. VO_2_ and energetic cost were measured using a portable metabolic system during the final minute of each trial. Subjective feedback was collected after each condition.

The key gait parameters (i.e., stride frequency, stride length, and contact time) were measured for each step by using an inertial measurement unit (RunScribe^TM^, Scribe Lab Inc., San Francisco, CA, United States) with a sampling frequency of 500 Hz. The RunScribe^TM^ device has shown excellent validity and adequate reliability for gait parameters measurement compared to optical measurement systems (Garcia-Pinillos et al., 2020; Lewin et al., 2022). According to the manufacturer’s instructions, the RunScribe^TM^ was attached to the instep of the participant’s both feet and paired with a mobile phone. The data was then analyzed using the RunScribe^TM^ Dashboard Center, available on the web. To ensure synchronization between gait and metabolic data, both the MetaMax 3B-R2 metabolic system and the RunScribe™ sensors were manually started and stopped simultaneously at the beginning and end of each 1600 m trial. This procedure ensured that gait and VO_2_ data corresponded to the same time intervals, from which the final 2 min were extracted for steady-state analysis. At the end of RE trial in each spike condition, participants anonymously completed a questionnaire assessing their subjective perceptions of the spikes condition, without knowledge of the corresponding measurement results. Specifically, they rated: (1) perceived overall comfort, (2) perceived cushioning, and (3) perceived performance enhancement (Lindorfer et al., 2019). Ratings were given on a 10-point Likert scale (1 = lowest, 10 = highest), with higher scores reflecting greater comfort, better cushioning, and more pronounced performance enhancement. llustration of the methods protocol of the present study as shown in Figure 2.

### 2.5 Statistical analysis

A repeated-measures analysis of variance was used to compare metabolic and running biomechanical data across three AFT spikes. The dependent variable was VO_2_, energetic cost, and spatiotemporal gait parameters, the independent variable was the AFT spikes condition (Dragonfly1, Dragonfly2, and Victory1). Descriptive statistics (Mean ± SD) for oxygen uptake, energetic cost and gait parameters of the dominant leg (defined as the preferred kicking leg) in the last 2 min of each condition. The normality of the data distributions was tested using the Shapiro–Wilk test, with p>0.05 indicating normality. Differences were analyzed using one-way repeated measures ANOVA with Greenhouse-Geisser correction (Greenhouse and Geisser, 1959), followed by a post-hoc pairwise comparison of the least significant difference for multiple comparisons (Holm, 1979) if a significant main effect was found. The significance level was set at 0.05 ( α = 0.05) to minimize the probability. The effect size for ANOVA was calculated using partial eta-squared ( $\eta_{p}^{2}$ ) and considered a small effect (0.01 ≤ $\eta_{p}^{2}$ < 0.06), medium effect (0.06 ≤ $\eta_{p}^{2}$ < 0.14), and significant effect ( $\eta_{p}^{2}$ ≥ 0.14) (Cohen, 2013). Due to the different pacing groups, no cross-group statistical comparisons were made; all conclusions are based on comparisons between AFT models within the same group.

In addition, perceived responses to the survey questions were analyzed using nonparametric Friedman tests, followed by post-hoc Wilcoxon signed-rank tests for paired comparisons (Zimmerman and Zumbo, 1993). The statistics were calculated using SPSS 26.0 (IBM Corporation, Armonk, NY, United States).

## 3 Results

During the RE assessment session, there was no significant effect for spike condition on running speed ( p>0.05 ), with the mean running speeds were 16.2 ± 0.5 km·h^−1^ for trained runners and 18.3 ± 0.6 km·h^−1^ for national-level runners. The respiratory exchange ratio (RER) remained below 1.0 throughout all trials, confirming submaximal steady-state exercise, whit mean RER values were 0.90 ± 0.03 for trained runners and 0.88 ± 0.05 for national-level runners. To evaluate whether the order of testing affected metabolic outcomes, a paired-samples t-test was performed comparing oxygen uptake between the first and third running trials, irrespective of footwear condition. The analysis revealed no significant difference between trials ( p>0.05 ), indicating that neither fatigue nor adaptation systematically influenced RE across the testing sequence.

**FIGURE 3:**
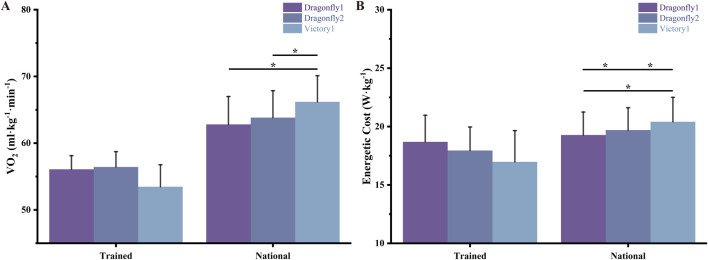
Oxygen uptake and energetic cost across three AFT spike conditions in trained and national-level runners. **(A)** Oxygen uptake (VO_2_, mL·kg^−1^·min^−1^) and **(B)** energetic cost (W·kg^−1^) during submaximal running trials under three footwear conditions: Dragonfly1, Dragonfly2, and Victory1. Data are shown separately for trained runners (left panels) and national-level runners (right panels). Error bars represent standard deviations and asterisks denote significant differences.

**FIGURE 4:**
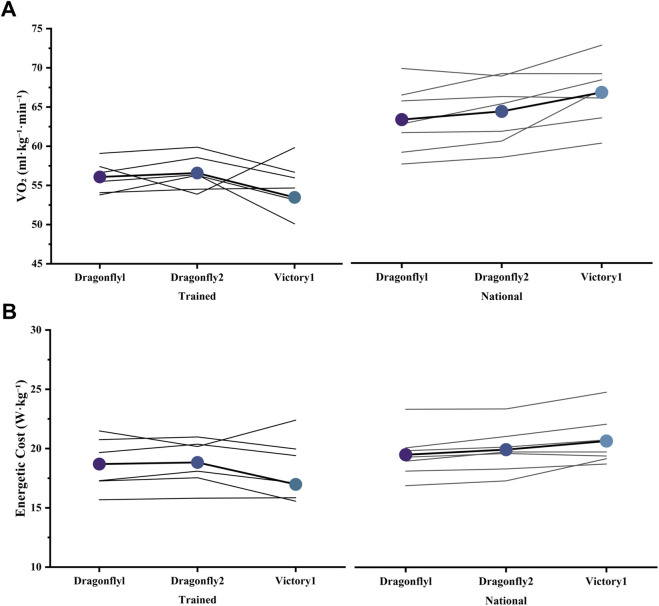
Individual oxygen uptake and energetic cost responses across three AFT spike conditions in trained and national-level runners. **(A)** Oxygen uptake (VO_2_, mL·kg^−1^·min^−1^) and **(B)** energetic cost (W·kg^−1^) during submaximal running under three footwear conditions (Dragonfly 1, Dragonfly 2, Victory 1). Data are shown separately for trained runners (left panels) and national-level runners (right panels). Each thin line represents an individual runner’s response across footwear conditions, and bold colored circles denote group means.

VO_2_ (ml·kg^−1^·min^−1^) and energetic cost (in W/kg) across AFT spike conditions are expressed in Table 2 and Figures 3, 4. Running mechanics data across spike conditions are also expressed in Table 2. Supporting H1, significant effects of AFT spike conditions were found for national-level runners ( p<0.05 ; $\eta_{p}^{2}=0.616$ ) and energetic cost ( p<0.05 ; $\eta_{p}^{2}=0.651$ ), but not for the trained runners ( p>0.05 ; $\eta_{p}^{2}=0.128$ for RE and 0.099 for energetic cost). In national-level athletes, VO_2_ and energetic cost were significantly higher in the foam-plus-plate condition (Victory1) compared with both foam-only models (Dragonfly1 and Dragonfly2, p<0.05 ). confirming that foam-only spikes elicited better RE. Furthermore, energetic cost was also greater in Dragonfly2 than Dragonfly1 ( p<0.05 ).

**TABLE 2:** Comparison of independent variables for different types of AFTs during the RE tests (i.e., descriptive statistics and one-way repeated measures ANOVA outcomes of RE and gait parameters in different AFT conditions).

	Dragonfly1	Dragonfly2	Victory1	p	F	$\eta_{p}^{2}$
Trained						
VO_2_ (mL/kg/min)	56.08 ± 2.04	56.43 ± 2.30	53.47 ± 3.30	0.449	0.737	0.128
Energetic cost (W/kg)	18.69 ± 2.28	17.95 ± 2.01	16.98 ± 2.67	0.516	0.549	0.099
Step frequency (steps/min)	185.49 ± 6.86	185.19 ± 7.47	182.31 ± 7.94	0.723	0.190	0.037
Stride length (m)	2.933 ± 0.101	2.911 ± 0.048	2.983 ± 0.166	0.627	0.321	0.060
Contact time (ms)	213.25 ± 10.24	213.29 ± 9.59	209.43 ± 7.25	0.297	1.376	0.216
National						
VO_2_ (mL/kg/min)	62.81 ± 4.18^c^	63.83 ± 4.03^c^	66.19 ± 3.92^a,b^	<0.01	9.627	0.616
Energetic cost (W/kg)	19.28 ± 1.96^b,c^	19.70 ± 1.91^a,c^	20.41 ± 2.09^a,b^	<0.01	11.212	0.651
Step frequency (steps/min)	186.89 ± 9.08	187.02 ± 7.46	187.54 ± 7.76	0.814	0.172	0.028
Stride length (m)	3.266 ± 0.238	3.278 ± 0.241	3.295 ± 0.201	0.571	0.508	0.078
Contact time (ms)	196.34 ± 9.76^b,c^	191.79 ± 6.84^a^	191.57 ± 6.83^a^	<0.05	4.275	0.461

Note that a, b, and c denote significant differences relative to Dragonfly1, Dragonfly2, and Victory1, respectively.

Relating to H2, analysis of gait spatiotemporal parameters revealed no significant main effect of AFT spike conditions in stride length ( p>0.05 ) and step frequency ( p>0.05 ), but a significant main effect on contact time was observed in the national-level groups ( p=0.046 ; $\eta_{p}^{2}=0.461$ ). Specifically, foam-plus-plate (Victory1) and foam-only (Dragonfly2) spikes showed shorter contact times than Dragonfly1( p<0.05 ), whereas no difference was observed between Dragonfly2 and Victory1 ( p>0.05 ).

Consistent with H3, significant effects of AFT spike conditions were found for perceived comfort ( p<0.05 ; $\eta_{p}^{2}=0.571$ ) and cushioning scores ( p<0.05 ; $\eta_{p}^{2}=0.265$ ) in both trained and national-level runners (as shown in Table 3). Participants rated the foam-only Dragonfly models as more comfortable (Dragonfly1: 8.08 ± 1.19; Dragonfly2: 8.38 ± 1.39; Victory1: 5.38 ± 2.26). and better cushioned (Dragonfly1: 8.15 ± 1.21; Dragonfly2: 7.38 ± 1.66; Victory1: 6.15 ± 2.64) than the foam-plus-plate Victory, with no difference between the two Dragonfly versions. However, there was no significant effect of AFT spikes condition on performance enhancement ( p=0.254 ; $\eta_{p}^{2}=0.108$ ; Dragonfly1: 7.85 ± 1.07; Dragonfly2: 7.77 ± 1.30; Victory1: 8.23 ± 0.93), indicating that subjective performance ratings did not differ across models.

**TABLE 3:** Subjective ratings of comfort, cushioning, and perceived performance enhancement (mean ± SD) across AFT spike models.

	Dragonfly1	Dragonfly2	Victory1	p	$\eta_{p}^{2}$
Perceived comfort	8.08 ± 1.19^c^	8.38 ± 1.39^c^	5.38 ± 2.26^a,b^	<0.01	0.571
Perceived cushioning	7.38 ± 1.66^2^	8.15 ± 1.21^a,c^	6.15 ± 2.64^b^	<0.05	0.265
Perceived performance enhancement	7.85 ± 1.07	7.77 ± 1.30	8.23 ± 0.93	0.254	0.108

Note that a, b, and c denote significant differences relative to Dragonfly1, Dragonfly2, and Victory1, respectively.

## 4 Discussion

This study compared two commercially available configurations of AFT spikes under submaximal, race-relevant conditions in trained and national-level distance runners. The principal finding was that among national-level athletes running at 18 km·h^−1^, foam-only spikes elicited lower oxygen uptake and energetic cost than the plate-integrated spike, whereas no differences in RE were detected among trained athletes running at 16 km·h^−1^. Specifically, we found that a plate-integrated spike model (Victory1) elicited significantly greater oxygen uptake and energetic cost compared to two foam-only models (Dragonfly1 and Dragonfly2), but only among national-level athletes with faster running speed. This result aligns with the findings of Carranza et al. (Rodrigo-Carranza et al., 2025), who reported that AFT spikes combining modern foam and carbon fiber plates performed 1.1% worse than spikes using only modern foam. Although previous studies have suggested that carbon plates may benefit short-distance or sprint performance(Nagahara et al., 2017), the current data indicate that such rigid components may increase energy expenditure at endurance speeds. Despite the theoretical mechanical advantages of carbon fiber plates, overly rigid footwear may negatively impact RE at submaximal running speeds (Oh and Park, 2017).

Previous studies have shown that metabolic cost decreases with increased LBS; however, the benefits do not persist indefinitely, indicating the presence of an optimal LBS (Oh and Park, 2017; Roy and Stefanyshyn, 2006). McLeod et al. (McLeod et al., 2020) identified an optimal stiffness of 19.29 N/mm at a running speed of 4.47 m/s. This may partly explain the lower energetic cost observed in national-level runners wearing the Dragonfly compared to the Victory in our study, as the LBS of the Victory (41.2 N/mm) exceeded that of the Dragonfly (Dragonfly1 of 12.4 N/mm and Dragonfly2 of 13.2 N/mm, respectively). However, as *in vivo* lower-limb stiffness and joint kinetics were not directly measured, these interpretations remain speculative and should be viewed as theoretical explanations consistent with prior literature rather than experimentally verified mechanisms. In this study, to ensure submaximal intensity during testing, the RE test speeds were set at 16 km/h (4.44 m/s) for the trained group and 18 km/h ((5.00 m/s)) for the national-level group, corresponding to approximately 85% of their respective 10,000-m race speeds. A known limitation of RE testing, however, is that it must be conducted at a sufficiently moderate pace to allow oxygen uptake to reach a steady state. As a result, even when the test speed is increased, it typically remains lower than the actual race pace achieved by competitive runners over 10,000 m. However, Hoogkamer et al. (Hoogkamer et al., 2016) demonstrated that differences in RE measured under submaximal conditions in the laboratory can reliably predict performance changes in 3000-m time trials performed at higher running speeds.

These findings partially align with prior work showing that AFT footwear can improve RE by enhancing mechanical efficiency through compliant midsoles and appropriately tuned stiffness. Notably, the trained group in this study was tested at 16 km·h^−1^, whereas the national-level group was assessed at 18 km·h^−1^, which could influence sensitivity to metabolic differences. However, the present results challenge the assumption that increasing LBS via a carbon-fiber plate universally enhances metabolic performance, particularly in elite runners during prolonged efforts. Our observations at 16 km·h^−1^ are consistent with previous data showing no RE difference between two top-performing AFT spikes (Dragonfly 1 vs. Adidas Avanti TYO) despite the inclusion of a carbon plate (Joubert et al., 2024). Taken together with evidence that AFT-related advantages tend to be larger at higher speeds and in highly trained runners (Stephen et al., 2025), these patterns suggest a speed- and level-dependent response, in which the net benefit depends on how plate-induced stiffness interacts with runner mechanics at endurance paces.

There has been some study that has shown that AFT shoes can improve RE (Hoogkamer et al., 2018; Rodrigo-Carranza et al., 2023) and long-distance performance (Senefeld et al., 2021; Rodrigo-Carranza et al., 2022). However, the impact of RE on running track events has been proven to be controversial and can vary depending on the race distance (Jones, 1998; Ingham et al., 2008). RE plays a relatively greater role in longer-distance events (e.g., 5000 m and 10,000 m) compared to middle-distance events (e.g., 800 m and 1500 m) (Lacour et al., 1990; Bellinger et al., 2021). Building on these findings, our study showed that at a submaximal speed of 5.0 m/s, RE was significantly better in the foam-only model compared to the plate-integrated one. Longer-distance track events such as the 5000 m and 10,000 m are typically run at average speeds around 5.4–5.7 m/s, which are slower than those of middle-distance events like the 800 m or 1500 m. Given this, our findings suggest that foam-only configurations may offer a performance advantage in longer-distance events where running speeds are relatively lower. Notably, improvements in 3000 m time trial performance were greater with carbon plates (1.0% without vs. 2.4% benefit with carbon plates), suggesting that the addition of a plate may become increasingly beneficial as race distance shortens and running speed increases (Rodrigo-Carranza et al., 2025).

Several studies have examined the variation of spatiotemporal parameters during AFT running, with step frequency, stride length and contact time being the most studied (Hoogkamer et al., 2018; Barnes and Kilding, 2019; Joubert et al., 2024; Rodrigo-Carranza et al., 2025). There were no significant effects of shoe condition in stride length ( p>0.05 ) and step frequency ( p>0.05 ). For contact time, we found significant effects for AFT spike condition for national groups ( p=0.046 ), but not for trained runners ( p>0.05 ). The contact time of Dragonfly2 and Victory1 were significantly lower than those of Dragonfly1, with no significant differences between Dragonfly2 and Victory1. A more curved, full-length, non-carbon plate creates a propulsive rocker-like (“teeter totter”) effect, which may accelerate the heel-to-toe flip, and in combination with a smaller heel-to-toe drop and higher energy return, this configuration may shorten the contact time. This reduction in contact time is not due to a single factor, but rather to the synergistic effects of the structural and material upgrades in the Dragonfly2: a more curved plate body creates the propulsive rocker effect, a smaller heel-to-toe drop, higher energy return, and a lighter weight mass.

Although gross measures of gait characteristics showed little difference between different spikes, a biomechanical explanation for energetic differences is important to consider. Previous research has shown that contact time is inversely related to metabolic rate: shorter contact times require faster muscle contractions to support body weight and thus increase energy expenditure (Kram and Taylor, 1990; Healey and Hoogkamer, 2022). Consistent with these findings, we found that the Victory spikes produced a significant reduction in contact time accompanied by an increase in energetic cost. However, contact time alone cannot fully account for the differences in RE across spike designs. Multiple interacting factors, including the LBS associated with the curved carbon-fiber plate, the viscoelasticity of the modern foams, shoe mass distribution, and potential changes in foot-strike pattern or ankle joint moment pathways, may collectively influence running energy metabolism.

Previous studies have shown that minimally cushioned shoes often shorten ground contact time (Gillinov et al., 2015). In contrast, a recent meta-analysis reported that increasing LBS generally prolongs contact time, presumably because a stiffer forefoot delays roll-over. Our data diverge from that pattern: in both trained and national-level runners, the stiffer spike (Victory) exhibited shorter contact times than its foam-only counterparts. One plausible explanation is that Victory’s higher LBS brings the foot-ground system closer to McMahon’s “tuned stiffness” region (McMahon and Greene, 1979), where leg stiffness and surface compliance are optimally matched, allowing faster energy transfer and thus briefer stance durations. Supporting this interpretation, sprint-specific research has shown that increasing forefoot stiffness can reduce joint loading around the metatarsophalangeal joint and simultaneously shorten contact time on the track (Hazman et al., 2023). This apparent discrepancy may reflect the difference between submaximal endurance running and sprinting mechanics. At the moderate speeds tested in this study, shorter contact time does not necessarily indicate greater mechanical efficiency but may instead reflect increased vertical loading or altered leg-spring dynamics that elevate metabolic cost. In contrast, at higher sprinting speeds, reduced contact time can facilitate more efficient elastic energy transfer. Thus, the observed relationship likely depends on running intensity and mechanical context rather than representing a universal effect of stiffness. Taken together, these findings suggest that when stiffness is tuned to an athlete’s mechanics and running speed, a carbon-plated spike can facilitate a quicker leverage phase despite its greater rigidity, whereas excessive or poorly matched stiffness would have the opposite effect. This discrepancy may stem from differences in athlete population, running speed, or spike configuration, indicating that the interaction between spike stiffness and contact mechanics is likely context-dependent and multifactorial. It should be noted, however, that these proposed mechanisms are inferential, as no joint kinetics or lower-limb stiffness measures were collected in the present study. Therefore, the role of LBS in mediating the observed metabolic differences should be interpreted as hypothetical rather than directly confirmed. Differences in contact time among spike models may be attributed to variations in forefoot structural properties. Dragonfly1, which exhibited the longest contact time, had the lowest forefoot LBS (13.4 N/mm) and lowest energy return (82.1%). In contrast, Victory1 showed the shortest contact time alongside the highest LBS (41.2 N/mm) and energy return (83.9%), which may partly explain the quicker force transfer and faster transition into swing phase. Although Dragonfly2 shared similar LBS with Dragonfly1, its slightly higher foam thickness and energy return could contribute to its intermediate contact time.

All runners in the present study perceived the foam-only spikes to be more comfortable $\left(p<0.05\right.$ ) than the foam combined with a carbon fiber plate design. This difference in comfort may be attributed to the increased LBS introduced by the carbon plate, as well as reduced forefoot cushioning, both of which may negatively influence perceived comfort. The higher comfort ratings observed for both Dragonfly models appear to track closely with their superior perceived cushioning scores. In our cohort, Dragonfly2 received the highest cushioning and comfort ratings, even though it shared similar LBS with Dragonfly1. In contrast, Victory, which had a stiffer forefoot and thinner foam, was judged to be the least cushioned and least comfortable. These findings suggest that perceived cushioning may act as a mediator between midsole construction and global comfort perception, providing a plausible subjective mechanism for the general preference toward foam-only spikes. Notably, enhanced comfort may be particularly relevant for long-distance track events, such as the 10,000 m, where sustained discomfort can negatively affect performance. Previous studies have suggested a moderate association between perceived comfort and RE (Van Alsenoy et al., 2023), with Luo et al. (Luo et al., 2009) reporting a nearly 0.7% improvement in RE at aerobic threshold intensities as comfort increased. However, this relationship should be interpreted with caution, as comfort and RE are correlated rather than causally linked, and substantial inter-individual variability may limit the generalizability of these findings. For instance, some evidence indicates that runners respond differently to midsole cushioning and surface compliance, with some experiencing metabolic benefits from softer, more compliant footwear, while others show better responses to firmer or lighter configurations (Franz et al., 2012; Tung et al., 2014). These observations support the view that both comfort perception and the optimal spike design are highly individualized, even among similarly trained runners. Previous research on AFT has shown that athletes often perceive spikes equipped with carbon fiber plates to offer greater performance enhancement compared to foam-only designs (Hébert-Losier et al., 2024). In line with these findings, the current study observed higher perceived performance enhancement scores for the Victory spike (foam plus plate) relative to the Dragonfly models (foam only). However, this difference did not reach statistical significance, which may reflect inter-individual variability in athlete responses to footwear characteristics. Furthermore, because participants were not blinded to the visually distinguishable spike models, perceptual ratings may have been influenced by expectancy effects, representing a potential source of bias.

The main objective of the present study was to compare the effects of two commercially available AFT spike models, Nike ZoomX Dragonfly (foam-only) and Nike Air Zoom Victory (foam combined with a carbon fiber plate), on RE in trained and national-level distance runners under submaximal running conditions. This comparative analysis demonstrates that the foam-only model tested (Dragonfly) yield superior RE compared with the carbon-plated model tested (Victory). While both spike configurations are used in competitive settings, the addition of a carbon fiber plate in the tested model did not provide additional metabolic advantages and may even increase energetic cost when shoe stiffness exceeds the optimal range. Subjective ratings consistently favored the foam-only spikes in terms of comfort, whereas perceived performance enhancement was similar between models. These findings should be interpreted as specific to the tested spike models, rather than generalized to all foam-only or carbon-plated footwear. Evidence-based, individualized spike selection is therefore essential to optimize performance across different track events.

The present study has several limitations. First, our study and the current analysis were limited to male runners. Although previous research has demonstrated comparable improvements in RE for both male and female participants when evaluating the effects of AFT (Joubert et al., 2023; Barnes and Kilding, 2019), future studies including female athletes are necessary to further validate and expand upon the effects of AFT spikes in female runners. Moreover, direct comparisons between groups were limited by the fact that the trained and national-level groups were tested at different speeds. Significant differences in RE with different spike structures were only observed in the national-level group, whereas no such effect was observed in the trained group. However, this absence of statistical significance in the trained group should be interpreted with caution, as the smaller effect sizes and limited sample size may have reduced the statistical power to detect meaningful differences. Future studies including larger samples are warranted to confirm whether similar trends exist among trained runners. These findings suggest that structural differences in AFT spikes may still affect RE in high-level athletes. Although previous studies have suggested that the performance advantage conferred by AFT footwear over conventional shoes may diminish with increasing levels of running, our findings suggest that this effect may be context-dependent and warrants further study in higher level populations. Furthermore, considering the lower relevance of RE to middle-distance track events, future research should incorporate middle-distance time-trial performance tests to more directly evaluate the comparative effectiveness of two spike designs in these events. Although each participant performed only one randomized trial per footwear condition, this approach is consistent with previous RE studies on advanced-footwear technology (Rodrigo-Carranza et al., 2023). Nevertheless, incorporating repeated and counterbalanced trials could further enhance internal validity in future research (Barrons et al., 2024). In addition, future investigations should integrate detailed kinematic and kinetic analyses, along with sex-based comparisons, to elucidate the biomechanical mechanisms underlying the effects of AFT spike designs on RE and performance across different race distances.

## Data Availability

The raw data supporting the conclusions of this article will be made available by the authors, without undue reservation.
